# STICK TO THE THEORY: FINDINGS FROM A COMPLEX CARE INTERVENTION FOLLOWING TRANSITION TO DIGITAL DELIVERY

**DOI:** 10.1093/geroni/igac059.1546

**Published:** 2022-12-20

**Authors:** Kylie Meyer, Carole White

**Affiliations:** Case Western Reserve University, Cleveland, Ohio, United States; UT Health San Antonio, San Antonio, Texas, United States

## Abstract

Two thirds of family caregivers to persons living with dementia provide complex care tasks, including medical/nursing tasks, and nearly half worry about making a mistake. Learning Skills Together (LST) was designed to prepare caregivers to provide complex care through hands-on instruction (e.g., practice using a gait belt). Consistent with self-efficacy theory, the in-person intervention integrated behavioral modeling, strengths-based feedback, and knowledge-building. COVID-19 prompted a transition to digital delivery of LST over Zoom. Intervention content was modified to accommodate a digital approach while continuing to adhere to self-efficacy theory. Results from a pre- and post-test pilot study (N=35) indicate improvement in self-efficacy on (mean difference (MD)=1.0, SD= 1.6, p-value=0.004). Caregiver comments during qualitative interviews affirm intervention objectives were met. For example, caregivers described the importance of peer learning (modeling) during discussion. Results indicate that complex care intervention can be digitally delivered to family caregivers to improve self-efficacy surrounding complex care.

